# The Effects of Temperature on Animal Gut Microbiomes

**DOI:** 10.3389/fmicb.2020.00384

**Published:** 2020-03-10

**Authors:** Juan Sepulveda, Andrew H. Moeller

**Affiliations:** Department of Ecology and Evolutionary Biology, Cornell University, Ithaca, NY, United States

**Keywords:** metagenomics, climate change, organismal biology, amplicon sequence variants (ASVs), operational taxonomic unit (OTU)

## Abstract

Temperature is a prominent abiotic environmental variable that drives the adaptive trajectories of animal lineages and structures the composition of animal communities. Global temperature regimes are expected to undergo rapid shifts in the next century, yet for many animal taxa we lack an understanding of the consequences of these predicted shifts for animal populations. In this review, we synthesize recent evidence that temperature variation shapes the composition and function of animal gut microbiomes, key regulators of host physiology, with potential consequences for host population responses to climate change. Several recent studies spanning a range of animal taxa, including Chordata, Arthropoda, and Mollusca, have reported repeatable associations between temperature and the community composition and function of the gut microbiome. In several cases, the same microbiome responses to temperature have been observed across distantly related animal taxa, suggesting the existence of conserved mechanisms underlying temperature-induced microbiome plasticity. Extreme temperatures can disrupt the stability of alpha-diversity within the gut microbiomes individual hosts and generate beta-diversity among microbiomes within host populations. Microbiome states resulting from extreme temperatures have been associated, and in some cases causally linked, with both beneficial and deleterious effects on host phenotypes. We propose routes by which temperature-induced changes in the gut microbiome may impact host fitness, including effects on colonization resistance in the gut, on host energy and nutrient assimilation, and on host life history traits. Cumulatively, available data indicate that disruption of the gut microbiome may be a mechanism by which changing temperatures will impact animal fitness in wild-living populations.

## Introduction

Variation in ambient temperature is a ubiquitous feature of every environment on Earth which organisms must endure to ensure their own survival and reproduction. The frequency and magnitude of temperature fluctuations are expected to increase globally over the next century (Deser et al., 2012), in part as a consequence of human-induced climate change. As many as one in six animal species may be threatened with extinction by shifting temperature regimes (Urban, 2015), with potential cascading consequences for animal communities and wider ecosystems. Predicting biodiversity responses to changing temperature regimes first requires an understanding of the mechanisms by which temperature impacts organismal physiology and fitness.

Rising temperatures may negatively affect animal fitness directly through effects on physiology, but they may also reduce fitness by disrupting mutualisms between animals and other organisms. The effects of temperature on species interactions are well documented in symbiosis between Eukaryotes. Changes in temperature has been implicated in disrupting multi-species interactions among plants, insects, and birds in experimental solardomes (Buse et al., 1999). Similarly, warming temperatures have been associated with disruption of top trophic levels in marine systems (Kordas et al., 2011). Some of the most pervasive symbioses into which animals enter are those with bacteria and archaea (McFall-Ngai et al., 2013), motivating the need to investigate the effects of temperature on interactions with these species as well. Every animal species harbors microbial communities both in and on the body. Although some of these animal-associated microbial communities may be sparse, unstable, or of minimal functional significance for their hosts (Hammer et al., 2019), many contribute in fundamental ways to host phenotypes and fitness. The densest communities of microorganisms associated with animals typically reside in the gastrointestinal tract. In many animal lineages, the gut microbiota has become deeply integrated with host metabolic, immune, and neuroendocrine systems (Kau et al., 2011). In mammals and insects, for example, germ-free (i.e., axenic) hosts of some species display a range of phenotypic differences when compared to host reared in the presence of a gut microbiota (Neufeld et al., 2011; Ridley et al., 2012). In addition, gut-microbiota transplant experiments into axenic hosts have revealed that variation in the microbiota can generate variation in host phenotypes (Faith et al., 2014; Gould et al., 2018). Recently, this gut-microbiota driven variation in host phenotype has been implicated in the adaptive evolution of host populations (Rudman et al., 2019) and species (Moeller et al., 2019), further suggested that the presence of specific microorganisms in the gut is important for host fitness. As temperature regimes change globally, any effect that these changes have on the composition of animal gut microbial communities may alter their functions and lead to consequences for host phenotypes and fitness. Therefore, understanding how ambient temperature impacts the gut microbiota of animals may help predict future responses of animal lineages and communities to climatic change.

In this review, we synthesize recent literature investigating the effect of ambient temperature on the composition and function of the animal gut microbiota. We focus on experiments designed to test the effects of temperature, rather than retrospective studies, although insights into the effects of seasonality and latitude on the gut microbiota are emerging (Smits et al., 2017; Orkin et al., 2019). The broad application of culture-independent, high-throughput sequencing methods across the experiments reviewed here allows straightforward comparison of results, revealing several general trends that appear across the animal phylogeny as well as host clade-specific effects. In addition, we discuss several routes by which variation in the gut microbiota generated by temperature changes may affect animal phenotypes and fitness.

## Effects of Temperature on the Composition of the Animal Gut Microbiota

**FIGURE 1:**
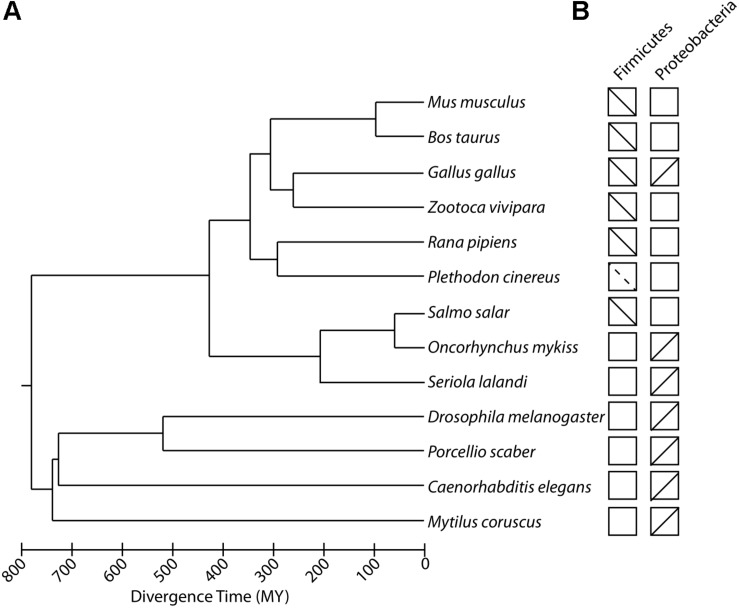
Experiments investigating effects of temperature on the composition of animal gut microbiomes. **(A)** Phylogenetic tree of animal species in which the effects of ambient temperature on the composition of the gut microbiota have been examined by experimental manipulations. Scale bar indicates divergence time in millions of years (MY). **(B)** Boxes correspond to host species at tips of phylogeny in **(A)** and contain lines indicating the relationships observed between temperature and the relative abundances of Firmicutes and Proteobacteria, two predominant gut bacterial phyla that display consistent trends across animal taxa. Upward and downward sloping lines indicate positive and negative associations within temperature, respectively. Dashed lines indicate instances in which temperature displayed consistent associations with relative abundances of genera within either Firmicutes or Proteobacteria.

Recent studies have shown that variation in temperature shapes the composition of the gut microbiota across the animal tree of life. In animals, the composition of the gut microbiota varies across taxa, and hosts from the same taxon often display more similar gut microbiota compositions than do hosts from different taxa (McFall-Ngai et al., 2013). Accordingly, the specific effects of temperature on the gut microbiota differ between animal taxa. Although many host species-specific trends have been reported, some general findings have emerged across animal clades. In particular, increases in temperature have been associated with changes in the community membership and relative abundances of specific bacteria (beta diversity) within the host individuals. Several recent studies further suggest that shifts in the gut microbiota in response to temperature may have cascading physiological consequences for host performance under different thermal conditions. In this section, we synthesize the compositional changes in the gut microbiota that have been reported in response to temperature in diverse animal taxa (Figure 1). We focus our synthesis on animal gut microbiotas, although it should be noted that recent studies have also reported temperature-induced shifts in animal microbiotas inhabiting other body sites, such as the hemolymph (Lokmer and Wegner, 2015) and epidermis (Fan et al., 2013). In the case of animal gut microbiotas, available data suggests that each host species displays a distinct microbial response to thermal stress, but some gut bacterial taxa, in particular lineages of Firmicutes and Proteobacteria, display consistent shift with temperature that appear to be reproducible across host species.

### Experiments in Vertebrates

The composition of the gut microbiota in vertebrates tends to be dominated by the phyla Firmicutes, Bacteroidetes, Actinobacteria, Proteobacteria, and Fusobacteria, although the relative proportions of these phyla differ among vertebrate lineages (Ley et al., 2008). In mammals, Firmicutes and Bacteroidetes dominate, whereas Proteobacteria comprise a larger fraction of the gut microbiota in many birds, reptiles, and fish. Recent studies have assayed the gut microbiota of lab-reared or enclosure-reared vertebrates at different temperatures (Tajima et al., 2007; Chevalier et al., 2015; Kohl and Yahn, 2016; Bestion et al., 2017; Fontaine et al., 2018; Zhu et al., 2019). Despite the differences in composition among the gut microbiotas of vertebrate lineages, several general trends in responses of the gut microbiota to temperature variation have been observed across host species.

In tetrapods, temperature has been shown to induce reductions in the relative abundances of the gut bacterial phylum Firmicutes. In mammals, this effect has been reported in mice and cows. Rearing house mice (*Mus musculus domesticus*) at 6°C lead to a reproducible shift in the composition of the gut microbiota marked in part by a reduction in the relative abundances of Firmicutes not observed in mice reared at 25°C (Chevalier et al., 2015). Conversely, rearing cattle (*Bos taurus*) at 20, 28, and 33°C led to a progressive decrease in the relative abundances of Firmicutes within the gut microbiota (Tajima et al., 2007).

Similar effects of temperature have also been experimentally observed in amphibians and reptiles, including, lizards, chicken, tadpoles, and salamanders. Populations of the common lizard (*Zootoca vivipara*) reared at maximum daily temperatures of 29.3 ± 0.4°C, 31.5 ± 0.5°C, and 32.1 ± 0.6°C displayed a progressive decrease in the relative abundances of Firmicutes with increasing temperature (Bestion et al., 2017). A similar effect has also been observed in tadpoles (*Lithobates pipiens*), which displayed decreases in relative abundances of Firmicutes at 28°C relative to at 18°C (Kohl and Yahn, 2016). The gut microbiotas of Salamanders (*Plethodon cinereus*) were also found to display progressive decreases in the relative abundances of genera within the Firmicutes (e.g., *Anaerotrucus*) at 10, 15, and 20°C (Fontaine et al., 2018). In addition, laying hens exposed to heat stress have been shown to exhibit significant decreases in the relative abundance of Firmicutes within the fecal microbiota (Zhu et al., 2019).

The mechanisms underlying the negative association between Firmicutes relative abundances and temperature in the tetrapod gut microbiota remain unclear. This association is particularly interesting, because it spans both endothermic and ectothermic host taxa. Characterization of the spatial structure of bacterial taxa within the gut microbiota has revealed that Firmicutes are overrepresented near host epithelia relative to the center of the lumen, at least in some mammals (Earle et al., 2015). One possibility is that animal hosts may invest metabolically in maintaining commensal and beneficial lineages of Firmicutes within the gut microbiota, and that divesting resources to cope with thermal stress may therefore lead to reductions in abundances of this phylum.

In addition to decreases in the relative abundances of Firmicutes within the Amniota gut microbiota, several studies have also observed an overall decrease in alpha diversity in response to higher temperatures. In mice, temperatures of 6°C were associated with higher phylum-level alpha diversity within the gut microbiota (Chevalier et al., 2015). Similarly, in lizards (Bestion et al., 2017), chickens (Zhu et al., 2019), and salamanders (Fontaine et al., 2018), higher temperatures were associated with a decrease in 16S operational taxonomic unit (OTU)- or amplicon sequence variant (ASV)-level alpha diversity within individual microbiota. In contrast, no significant associations between temperature and alpha diversity were observed in cows or tadpoles (Tajima et al., 2007; Kohl and Yahn, 2016).

The effects of ambient temperature on the gut microbiota have also been examined in fish species. Negative associations between rearing temperature and Firmicutes relative abundances have been observed in rainbow trout (*Oncorhynchus mykiss*) (Huyben et al., 2018). However, rearing temperature and the relative abundances of Firmicutes do not appear to be consistently associated with one another in all fish species, which typically harbor lower relative abundances of Firmicutes than tetrapods and higher relative abundances of Proteobacteria (Ley et al., 2008). Accordingly, many of the compositional changes in the fish gut microbiota in response to temperature variation that have been reported are driven by shifts in the relative abundances of Proteobacteria linages. For example, a recent study comparing the gut microbiotas of salmon (*Salmo salar*) found that increasing temperatures were associated with shifts in the relative abundances of Gammaproteobacteria linages, with decreases in the relative abundances of Acinetobacter and increases in the relative abundances of Vibrio species known to display pathogenic properties (Neuman et al., 2016). Similar shifts in Gammaproteobacteria abundances have been observed in yellowtail kingfish (*Seriola lalandi*) (Soriano et al., 2018).

### Experiments in Invertebrates

The composition of the gut microbiota varies substantially across invertebrate animal species but tends to display higher relative abundances of Proteobacteria compared to gut-microbiota composition in vertebrates. Variation in ambient temperature has been associated with changes in the composition of the gut microbiota in diverse invertebrate lineages, including both arthropods and molluscs.

In insects, increases in temperature have been associated with increased relative abundances of Proteobacteria. Developmental temperature has been shown to impact the composition of the gut microbiota of fruit flies (*Drosophila melanogaster*), with higher temperatures (31°C) leading to increased abundances of *Acetobacter*, a genus of Proteobacteria, relative to lower temperatures (13°C) (Moghadam et al., 2018). Similarly, the gut microbiotas of wood lice (*Porcellio scaber*) exhibited decreased relative abundances of Actinobacteria and increased relative abundances of Proteobacteria in response to increases in temperature (Horváthová et al., 2019). Effects of temperature on the relative abundances of Proteobacteria lineages have also been observed in worms (*Caenorhabditis elegans*): worms reared at higher temperatures displayed increases in the relative abundances of Agrobacterium, a genus of Proteobacteria, and a corresponding decrease in the relative abundances of Sphingobacterium, a genus of Bacteroidetes (Berg et al., 2016). Intriguingly, the effect of temperature on the relative abundances of these bacterial genera within worm hosts displayed the opposite sign of the effect of temperature on the relative abundances of these genera in the soil, suggested interactions between bacteria and hosts influence the effects of temperature on bacterial abundances. In contrast to insects and *C. elegans*, the positive association between Proteobacteria relative abundance and temperature has not been observed in molluscs. In mussels (*Mytilus coruscus*), temperature was not significantly associated with changes in the relative abundances of bacterial phyla, but instead with shifts in the relative abundances of several bacterial genera (Li et al., 2018). In particular, mussels exposed to higher temperatures displayed decreases in the relative abundances of several bacterial genera and an overall decrease in alpha diversity.

### Field Studies

In addition to testing directly the effects of temperature on the animal gut microbiota in controlled experimental conditions, several recent studies have identified associations between temperature and the composition of the gut microbiota in wild-living hosts. These studies are limited in their ability to identify effects of temperature on the gut microbiota by the fact that temperature often co-varies with other environmental variables that can affect gut-microbiota composition, such as food availability. However, these studies are essential to determine how temperature effects on the gut microbiota are realized in natural host populations. To date, most field-based studies of associations between temperature and the animal gut microbiota have focused on comparing gut-microbiota compositions among animal hosts across temperature gradients along both temporal (e.g., seasonality) and spatial (e.g., altitude, latitude) axes.

Changing seasons have been shown to dramatically reshape the gut microbiota in some animal clades. However, these shifts in the gut microbiota are thought to primarily reflect differences in host diet between seasons rather than shifts in temperature, and the specific changes in the gut microbiota found to be induced by temperature in animal experiments (e.g., Firmicutes relative abundance decreasing with temperature) are often not observed between seasons in wild-living hosts. For example, wild mice display differences in gut microbiota composition between seasons marked by shifts in the relative abundances of bacterial taxa and functions related to seasonal differences in the availability of dietary items (Maurice et al., 2015). Similar results have been observed in human populations of hunter gatherers (Smits et al., 2017). Seasonality has also been shown to affect the composition of the microbiome in Galapagos *Geospiza fuliginosa* and *Geospiza fortis* finches, which harbored higher abundance of gammaproteobacterial in dry seasons and an overrepresentation of Actinobacteria and Bacilli in wet seasons (Michel et al., 2018). In addition, the gut microbiotas of oysters have also been shown to change cyclically with seasons, displaying reduced alpha diversity in winter months (Pierce et al., 2016).

In many animal taxa, seasonal shifts in temperature induce behavioral and physiological changes, such as hibernation, which in some cases have been associated with changes in the composition of the gut microbiota. For example, the composition of the gut microbiota has been shown to be associated with hibernation in bees (Bosmans et al., 2018), ground squirrels (Carey and Assadi-Porter, 2017), and bears (Sommer et al., 2016). In bears, gut-microbiota transplant experiments have further shown that the summer-associated bear gut microbiota confers increases in adiposity in germ-free mice. These results suggest that seasonal shifts in the gut microbiota may contribute to adaptive phenotypic plasticity in their hosts.

In addition to seasonality, several studies have reported associations between spatial variation in temperature and the composition of the gut microbiota. For example, both fruit flies and humans display some evidence of differences in gut-microbiota composition with latitude (Walters et al., 2018; Rudman et al., 2019), although the degree to which these shifts are driven by temperature remains poorly understood. Similarly, high-altitude mammals display altered gut-microbiota composition (Zhang et al., 2016). These shifts may be driven in part by reduced temperatures, but they may also reflect other environmental factors associated with altitude, such as oxygen concentrations. Understanding how ambient temperature specifically contributes the composition of the gut microbiota in wild-living populations of animals remains an exciting area for future research.

## Functional Consequences of Temperature-Induced Shifts in Animal Gut Microbiomes

Changing the community composition of the gut microbiota can alter its functional properties, thereby affecting host phenotypes and potentially fitness. The widespread effects that temperature has on the gut microbiota composition of diverse animal lineages invite questions regarding how this gut microbiota plasticity affects animal individuals and populations. In this section, we outline several routes by which changes in the composition of the gut microbiota in response to temperature may affect host fitness. In particular, we highlight recent experiments that have demonstrated a causal relationship between temperature-associated changes in the gut microbiota and host performance. Temperature induced changes in the gut microbiota can be deleterious for hosts, but they may also serve as cues that contribute to adaptive host phenotypic plasticity.

### Nutrient Assimilation and Host Metabolism

A primary function that the gut microbiota provides to animal hosts is increased digestive efficiency of complex polysaccharides and other molecules that are otherwise inaccessible to animal metabolism. Therefore, alterations of the gut microbiota caused by temperature may affect the metabolic costs and benefits received by hosts. In extreme cases of tight-knit symbiosis that are essential for host metabolism, such as insects and their bacterial symbionts, effects of extreme temperatures on host survival may be mediated by disruption of microbial associations (Zhang et al., 2019). However, the degree to which complex gut microbiotas mediate effects of temperature on host nutrient assimilation and metabolism is only beginning to be explored.

Several studies have reported associations between changes in the composition of the animal gut microbiota in response to temperature and changes in host digestive performance and metabolism. Fontaine et al. (2018) reported a decrease in energy assimilation, food intake, and digestive efficiency in salamanders reared at temperatures different from the host’s preferred temperature, and these changes in energy flux parameters were associated with specific changes in the microbiome composition. In particular, energy assimilation was associated with the relative abundances of the genera *Sphingopyxis* and *Roseococcus* as well as the genus *Stenotrophomonas*, which contains lineages capable of digesting cellulose polymers (Dantur et al., 2015). Similarly, in cattle, warm temperatures were associated with compositional shifts in the gut microbiota as well as decreased digestive performance in hosts, measured by rate of dry-mass digestibility (Tajima et al., 2007). However, in both of these cases, it remains unclear whether the changes in the gut microbiota in response to temperature are responsible for decreased host energy acquisition from the diet, as opposed to both trends reflecting other effects of temperature on host physiology. Differentiating between these competing hypotheses will require direct experimental manipulation of microbiota composition within hosts.

In mice, microbiota transplant experiments into germ-free hosts afford opportunities to directly interrogate the effects of temperature-induced changes in gut-microbiota composition on host phenotypes and fitness. To date, such experiments have been performed with regard to cold-induced changes in gut-microbiota composition (Chevalier et al., 2015) but not with regard to the effects of heat stress (although bacterial endosymbionts have been shown to affect host heat tolerance in insects; Zhang et al., 2019). Chevalier et al. (2015) transplanted the compositionally distinct gut microbiotas of mice reared in cold and room temperatures into germ-free mice and observed metabolic host responses. This experiment revealed that the cold-associated gut microbiota induced white-fat browning and elevated metabolic rate in mice. Further experiments have shown that cold-induced increases in bile acids contribute to cold-induced compositional shifts in the mouse gut microbiota (Worthmann et al., 2017), and that elimination of the microbiota impairs thermogenesis via brown adipose tissue (BAT) and reduces host energy metabolism (Li et al., 2019). Similar effects of cold temperatures have been observed in fruit flies, which exhibit gene-expression changes in the gut in response to cold driven by shifts in the gut microbiota (Zare et al., 2018). Overall, these experiments provide evidence that temperature-induced changes in the gut microbiota can have fast-acting metabolic consequences for hosts. In addition, these results are consistent with a history of host adaptation to the plastic responses of its gut microbiota. Specifically, hosts appear to have evolved to recognize cold-associated shifts in the gut microbiota and adjust metabolic processes accordingly.

### Colonization Resistance

Beyond contributions to host metabolism, a primary role of the gut microbiota for hosts is providing protection against infection by pathogens. The gut microbiota at once guides the development of the host immune system and occupies ecological niches in the gut that may otherwise be available to pathogens. Disruption to the gut microbiota can therefore deleteriously affect host fitness by way of eliminating these beneficial functions. For example, immunocompromised individuals often display an altered gut microbiota (Lozupone et al., 2013; Moeller et al., 2013), which in some cases may contribute to systemic infection and host mortality (Barbian et al., 2018). Although the effects of temperature-induced changes in the gut microbiota on host colonization resistance have not been established, several recent lines of evidence suggest that disruption of animal gut microbiota by temperature may reduce the resistance of hosts to invasion and colonization by microorganisms with pathogenic qualities.

Heat stressed individual hosts may be more likely to harbor by diverse microbial lineages not typically found at appreciable abundances within the gut microbiota, as evidenced by the increased compositional heterogeneity of the gut microbiota among individuals reared under stressful thermal conditions. Several studies have reported increased beta-diversity among the gut microbiota of hosts reared at temperatures approaching thermal maxima relative to hosts reared at more optimal temperatures. For example, a recent study of the gut microbiota of tadpoles found that hosts displayed increased beta-diversity in the gut microbiota among hosts when reared at warm temperatures compared to cooler temperatures (Kohl and Yahn, 2016). In addition, warm-reared hosts tended to harbor higher relative abundances of *Mycobacterium*, a genus with pathogenic representatives. Similar effects of thermal stress on compositional variation in the gut microbiota among individuals have been observed in corals, which harbor more compositionally heterogeneous microbial communities at warmer temperatures relative to cool temperatures (Zaneveld et al., 2017). The coral pathogen *Vibrio coralliilyticus* has been shown to display higher virulence at temperatures above 27°C than at cooler temperatures. A similar effect of heat stress on the colonization resistance of individual gut microbiota has been hinted at by a recent study of mussels, which found that heat-stressed individuals harbored greater relative abundances of potentially pathogenic lineages within the genera *Bacteroides* and *Acrobacter* (Li et al., 2018). Moreover, changes in the mussel microbiota induced by heat stress were associated with mussel mortality and also detected in mussels that suffered mortality regardless of rearing temperature, indicating that changes in the mussel gut microbiota in response to heat stress may contribute to host mortality.

Disruption of the gut microbiota may reduce colonization resistance by opening up niche space in the gut for pathogenic microorganisms, but it may also reduce colonization resistance by eliminating or reducing the abundances of gut bacteria that actively inhibit the growth of pathogenic microorganisms. For example, the wood louse *P. scabber* harbors antibiotic producing Actinobacteria in the gut whose abundance and diversity within hosts may affect host colonization resistance. Horváthová et al. (2019) showed that the relative abundances of lineages of Actinobacteria in *P. scabber* decreased with increasing temperatures. However, the relative contributions of increased ecological opportunity and decreased inter-microorganism competition to the invasibility of disrupted gut microbiota remain unclear.

In addition, disruption of the gut microbiota by extreme temperatures may decreased colonization resistance by way of negative impacts on the maturation and functioning of immune system, especially if the disruption occurs early in the animal’s life. The precise critical windows for microbial roles in host immunological development are still under investigation, but it is becoming increasingly clear that early life exposures to the microbiota guide the differentiation of immunological cells and tissues in the gut that protect hosts against infection. For example, in mice, the presence of a mouse-specific gut microbiota is required for complete differentiation of T-cell populations not seen when mice harbor a human- or rat-derived microbiota (Chung et al., 2012). If disruption of microbiota by temperature and loss of beneficial diversity is inherited in hosts, as has been observed in experiments of dietary fiber restriction (Sonnenburg et al., 2016), then host generations following extreme temperature events may lack exposure to microorganisms necessary for proper immunological development. Under this scenario, the negative fitness consequences of temperature mediated by the microbiota could amplify over host generations.

### Host Life History Traits

In addition to effects on host metabolism and immunity, changes in the gut microbiota can have downstream consequences for host life history traits, key components of fitness. Recent strain-inoculation experiments in axenic fruit flies (*Drosophila melanogaster*) have demonstrated that the presence of different combinations of gut bacterial strains within hosts shapes key host life history traits, including fecundity, development time, and time to death (Gould et al., 2018). Similarly, germ-free mice exhibit a range of changes in growth rate and fecundity-related phenotypes (Dubos and Schaedler, 1960). In addition, the host-species specific microbiota appears to be a key regulator of growth phenotypes, as house mice (*Mus musculus domesticus*) inoculated with the gut microbiota of closely related host species display altered growth curves than when inoculated with their own gut microbiota (Moeller et al., 2019). These results suggest that disruption of the gut microbiota by thermal stress could ultimately affect host growth and reproductive traits, but this hypothesis has not been tested with experiment.

A recent study in *D. melanogaster* provides some evidence that temperature induced shifts in the gut microbiota may affect host life history traits and fitness. The gut microbiota of *D. melanogaster* exhibits clinal variation in the relative abundances of acetic-acid bacteria and lactic-acid bacteria. These same bacteria have been shown through inoculation experiments with axenic flies to affect traits including developmental rate, lipid metabolism, and starvation thresholds. Inoculation of different proportions of these strains into fly populations elicited divergent rapid evolutionary responses in hosts, driving shifts in allele frequencies that mirror clinal variation in allele frequencies observed in wild populations (Rudman et al., 2019). These results provide strong evidence that variation in microbiota composition is contributing to adaptive divergence between wild fly populations. However, the extent to which clinal differences in bacterial abundances are driven by temperature, as opposed to other factors that vary with latitude, remains unclear. For example, while some of the clinal variation in the *D. melanogaster* gut microbiota may be driven by temperature, experiment evidence suggests that fly populations at different latitudes may be adapted to select for bacteria that confer host life history traits that improve fitness in the hosts’ respective geographic locations (Walters et al., 2018).

There is also emerging evidence that selection on hosts for cold tolerance may lead to shifts in the gut microbiota. A recent experimental evolution study of Tilapia found that artificially selecting hosts based on their cold tolerance generated compositional changes in the gut microbiota, and that cold-selected fish harbored gut microbiotas that were more resilient to cold than were fish reared and standard temperatures (Kokou et al., 2018). These results are consistent with contributions of the gut microbiota to host fitness in cold temperatures. However, the effects of cold-selected gut microbiota on Tilapia life history traits and other phenotypes have yet to be determined.

In tetrapods, changes in the gut microbiota driven by temperature have been associated with host lifespan, but the causal relationship between the gut microbiota and host lifespan in the context of temperature variation has yet to be established. A recent study of the common lizard (*Zootoca vivipara*) found that increasing temperatures by ~2°C led to a decline in alpha diversity within the gut microbiota of individual lizards, and that alpha diversity was negatively associated with host mortality (Bestion et al., 2017). These results are consistent with the possibility that temperature increases induced losses of commensal and beneficial bacterial diversity from lizard hosts, which in turn led to increased mortality. However, an alternative explanation is that both changes in microbiota alpha-diversity and increased mortality resulted from other factors, such effects of temperature on host physiology not mediated by the microbiota.

## Future Directions

Available evidence strongly suggests that temperature variation structures the composition and function of gut microbiomes in animals. One major outstanding question is the relative contributions of direct and indirect effects of temperature on the animal gut microbiome. Temperature is a major determinant of microbial diversity in microbiomes globally (Thompson et al., 2017), and therefore may directly alter gut microbiomes in animals, especially in those that are unable to precisely thermoregulate (e.g., ectotherms). However, temperature may also have indirect effects on the gut microbiome mediated by host responses. For example, temperature stress is known to affect host metabolism and energy budgets of a diversity of animals (e.g., Sokolova et al., 2012), which could in turn affect host investment in regulating microbiome composition. Teasing apart these direct and indirect effects of temperature on animal gut microbiomes represents an exciting area for future research.

**FIGURE 2:**
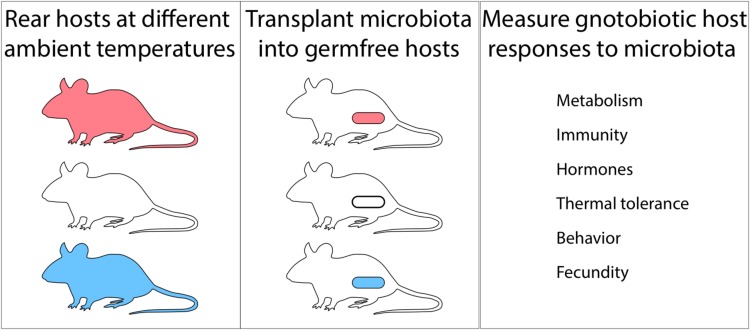
Germ-free animals provide avenues for discovering microbiota-mediated effects of temperature on hosts. Rearing hosts at different ambient temperatures **(Left)**, transplanting the hosts’ microbiota into germ-free animals **(Center)**, and measuring responses in gnotobiotic recipients **(Right)** can identify effects of changes in the microbiota driven by ambient temperature on host phenotype. Experiments in mice have shown that cold-driven changes in the gut microbiota cause responses in host metabolism that improve host cold tolerance (Chevalier et al., 2015).

Other major outstanding questions include whether and how shifts in the gut microbiome in response to temperature feedback to affect host phenotypes and fitness. While several studies have observed associations between temperature-induced variation in the gut microbiota and host phenotypes and fitness, few have demonstrated a causal role of the microbiota in these effects. A notable exception is a recent study that employed microbiota transplant experiments in germ-free animals to demonstrate that cold-induced shifts in the mouse gut microbiota confer adaptive phenotypes in the context of cold stress (Chevalier et al., 2015). Similar experiments that transplant the gut microbiota from individuals reared at different temperatures into germ-free hosts and observe the phenotypic consequences in the recipients (Figure 2) to test the effects gut-microbiota compositions associated with temperature changes represent exciting avenues for future research. Germ-free animal models include mice, chicken, zebrafish (Rawls et al., 2006), drosophila, and others, all of which provide opportunities to discover the effects of temperature-generated microbiota variation on hosts. This diversity of host systems also affords opportunities to understand what types of animals (e.g., ectotherms vs. endotherms) are most resilient to gut microbiome variation generated by changes in ambient temperature. Similarly, it will be possible to explore whether animals that have evolved in the presence of highly variable or stable gut microbiomes differ in their resilience to temperature-induced microbiome variation. Developing a mechanistic understanding of how gut microbiota variation under shifting global temperature regimes will affect animal phenotypes and fitness may improve prediction of population-level responses to climatic change.

## Author Contributions

JS conceived the project and wrote the manuscript. AM wrote the manuscript.

## Conflict of Interest

The authors declare that the research was conducted in the absence of any commercial or financial relationships that could be construed as a potential conflict of interest.
